# Athletic protocol in non-cystic fibrosis-related bronchiectasis: rationale and design of the pilot ATHOS study

**DOI:** 10.3389/fmed.2025.1554300

**Published:** 2025-07-09

**Authors:** Francesco Bertuccio, Mitela Tafa, Denise Carabetta, Tiziano Gemelli, Giuseppe D’Antona, Oscar Crisafulli, Valentina Conio, Amelia Grosso, Annalisa De Silvestri, Angelo G. Corsico, Giulia M. Stella

**Affiliations:** ^1^Department of Internal Medicine and Medical Therapeutics, University of Pavia Medical School, Pavia, Italy; ^2^Unit of Respiratory Diseases, Department of Cardiothoracic and Vascular, IRCCS Policlinico San Matteo, Pavia, Italy; ^3^Interdepartmental Centre in Motor and Sport Activities, Sport Medicine Centre, University of Pavia, Pavia, Italy; ^4^Department of Public Health, Experimental and Forensic Medicine, University of Pavia Medical School, Pavia, Italy; ^5^SSD Biostatistics and Clinical Trial Center, Fondazione IRCCS Policlinico San Matteo, Pavia, Italy

**Keywords:** bronchiectases, exacerbation, athletic, quality of life, activity

## Abstract

Bronchiectasis is a chronic and heterogeneous respiratory condition, which is characterized by irreversible abnormal dilatation of the bronchial tree, chronic cough, copious sputum production, and increased risk of acute exacerbations that contribute to the development of chronic respiratory failure, poor exercise tolerance and, consequently, poor quality of life (QoL). A large amount of published data explore regarding the diagnostic approach, the clinical management, and the development of novel therapeutic strategies. Moreover, it is well-known that the exercise-training rehabilitation can be helpful in reducing disease deterioration and relieve symptoms. However, the effect of exercise in patients with non-cystic fibrosis-related bronchiectasis (NCFB) is scarce, and no athletic programs have been fully developed. Thus, the aim of the present study is to investigate the results obtained by administering a specific athletic/physical protocol to these patients. Among all patients affected by NCFB and followed in our Institution, those with the highest scores of performance status will be addressed to the Interdipartimental Centre in Motor and Sport Activities, Sport Medicine Centre, University of Pavia for a work protocol based on mesocycles of 3 times/week for 6 months. A patient-tailored active training regimen will be set up considering major complaints—endurance training for patients with dyspnea, strength training for patients with cough and difficult sputum expectoration, and a balanced aerobic and anaerobic training for patients with asthenia. To the best of our knowledge, the ATHOS study is the first perspective clinical trial, encompassing athletic programs for non-CF bronchiectasis patients, and rationale and *in itinere*, partial results will be presented, analyzed, and discussed in comparison to standard disease management.

## 1 Introduction

### 1.1 Overview on bronchiectasis

Bronchiectasis is a condition characterized by abnormal and permanent dilations of the airways with a diameter greater than 2 mm (generally the subsegmental bronchi) resulting from the destruction of the elastic and muscular component of their wall, accompanied by chronic hyperproduction of mucopurulent or frankly purulent secretions (1). In approximately 30% of cases, they occur bilaterally with main involvement in the lower lobes; in forms of secondary to cystic fibrosis, the involvement of the upper lobes prevails (2). The involvement of only one lung segment is, commonly, the consequence of a localized infectious process or bronchial obstruction (3). Overall, the third-fourth-order bronchi and bronchioles are mainly involved and appeared as dilated, tortuous, flaccid airway, partially obstructed by viscous or purulent exudate. Based on their morphological appearance in bronchography, the Reid (4) classification grouped bronchiectasis in cylindrical, saccular, and varicose (4, 5). Microscopically, a thickening of the bronchial wall can be identified together with intense inflammatory exudation of the walls and desquamation of the epithelium with areas of necrosis, ulceration, and micro-abscesses; pseudo-stratification of cylindrical cells and squamous metaplasia in residual epithelial tracts can also be found. Bronchial lumen filled with purulent, yellow-greenish exudate, sometimes is hemorrhagic. Dilation of bronchial arteries, formation of vascular loops, and opening of shunts between the bronchial and pulmonary circulation occur as well; in end-stage disease, fibrotic reaction with obliteration of the bronchioles is demonstrated (2). Bronchiectasis cannot be classified as a distinct pathological entity since it is acquired pathologies and is the final result of non-specific events that lead to the destruction of the bronchial wall. Diffuse bronchiectasis can be congenital: secondary to pulmonary dysplasia, secondary to genetic alterations (immunodeficiency as condition predisposing to infections), ciliary dyskinesias leading to impaired mucociliary clearance with stagnation of secretions (e.g., Kartagener’s syndrome (6)), alteration of the rheological characteristics of secreted (facilitated bacterial adhesion) mainly associated to cystic fibrosis cystic fibrosis (7), Young’s syndrome defined by the association of respiratory tract infectious episodes and azoospermia (8), rheumatological diseases, and inflammatory bowel diseases. In conclusion, the pathogenetic hypothesis involves mechanical origin of bronchial dilations by action of pulsion forces (bronchial obstruction) from the inside or traction forces (cicatricial phenomena) or from the outside agents on bronchial walls weakened by a chronic inflammatory process. Moreover, any etiological factor or predisposing cause which determines chronic inflammation with—alteration of mucociliary clearance, stagnation of secreted bacterial colonization, and chronic infection; neutrophils accumulation with release of proteolytic enzymes and oxidizing radicals, functional and structural damage to the bronchial wall (increased susceptibility to infections); and damage resulting from products of bacterial origin (with direct, pro-inflammatory, immune-invasive harmful mechanism). Respiratory function is not altered in localized forms. In diffuse forms, obstructive syndrome with significant impairment of the small airways, consequent air trapping, and obstacle to the drainage of secretions is detected by spirometry, whereas mixed dysventilation occurs if fibrotic alterations are present. Moreover, reduced diffusing capacity of the lungs for carbon monoxide (DLCO) is observed together with ventilation–perfusion mismatch. Hypoxemia with normo/hypocapnia is associated in early stage of disease, whereas hypercapnia can occur later. Although a deeper discussion on the therapeutic approaches against bronchiectasis goes beyond the scope of this study, the most significant strategies encompass on resolution or treatment of predisposing factors (prevention of bronchopulmonary infections, subcutaneous or intravenous administration of immunoglobulins in immunodeficient conditions, timely removal of any lesion/body foreign obstructing the bronchial system); targeted/reasoned therapy of lung infections (to avoid the arousal of antibiotic resistant microbial strains, broncho-instillations); and maintenance of adequate bronchial toilet. Thus, validated therapeutic approaches are: physiokinesitherapy; antibiotic therapy in exacerbations (in more advance cases, the opportunity of maintenance therapy should be evaluated according to multidisciplinary evaluation); therapy of cystic fibrosis; corticosteroids and anti-inflammatory therapy (selective neutrophil elastase inhibitors, “scavenger” substances); bronchial arteriography and possible embolization in case of recurrent hemoptysis; surgical lung resection should be taken into consideration in case of localized forms, whereas bilateral lung transplant in case of end-stage disease.

### 1.2 Non-cystic fibrosis-related bronchiectasis

**Figure 1:**
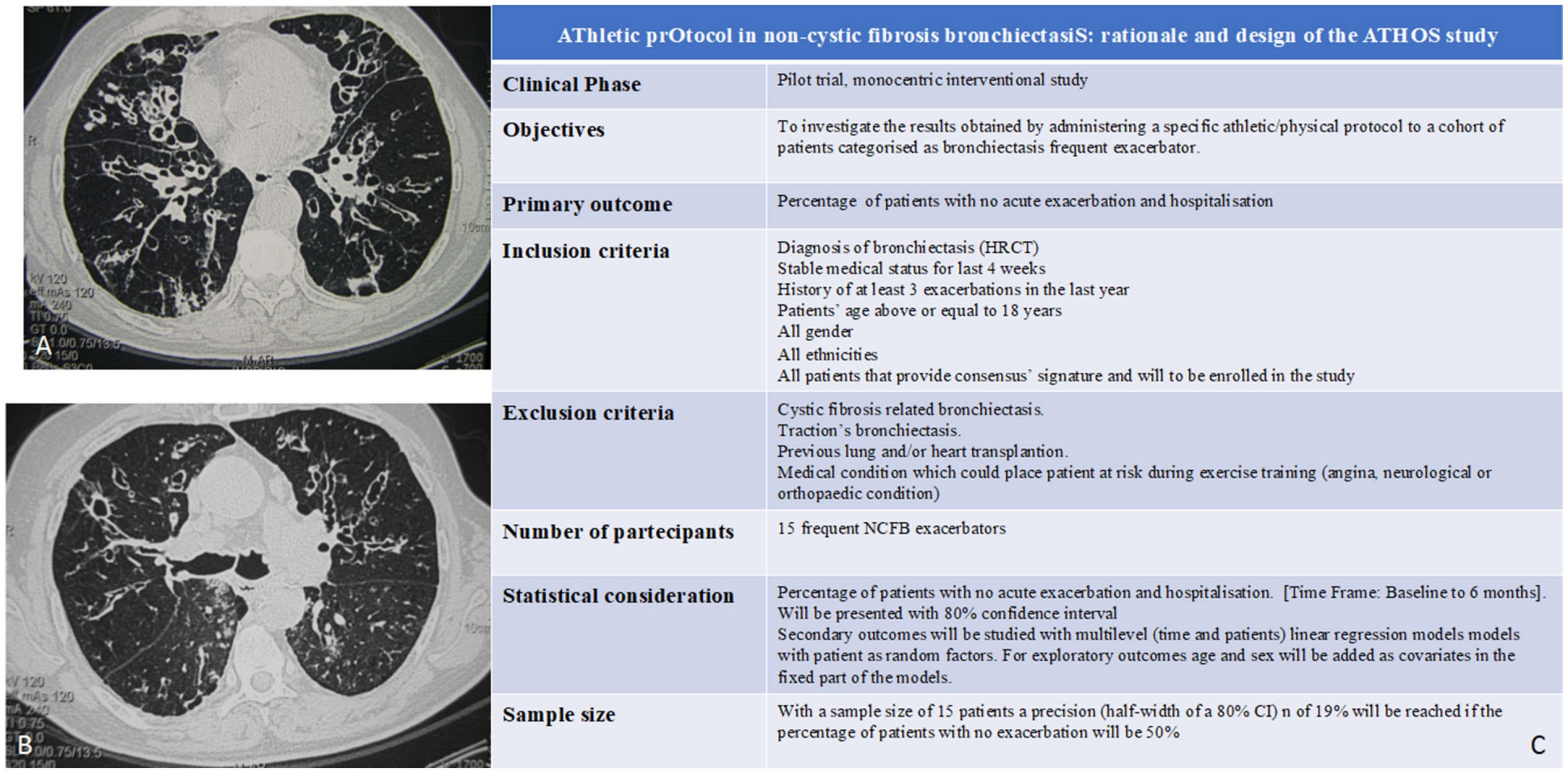
**(A,B)** CT scan features of bronchiectasis. Depending on the various causing factors, they can have local or widespread bilateral distribution. Predisposing factors are: bronchopulmonary infections, bronchial obstructions, congenital or acquired tracheobronchial pathologies, congenital alterations of the vascular and lymphatic system, congenital or acquired malformations of the chest wall and/or diaphragm, primary immune deficiencies, hereditary anomalies, *ab ingestis* pneumonia, inhalation of irritants, and systemic immune-inflammatory diseases (26). Localized bronchiectasis affects a segment or lobe of the lung and is the consequence of: bronchial obstructive process with downstream stagnation of secretions [such as foreign body aspiration, carcinoid (27), and adeno-bronchial syndrome (28)]; infectious processes [measles, whooping cough, respiratory syncytial virus, necrotizing pneumonia, and *Mycobacterium tuberculosis* (29–33)]; congenital or acquired anatomical defects; and retracting phenomena in fleshed tissue. Congenital bronchiectasis is related to malformations due to arrested development at the level of the lobar and segmental bronchi and the distal bronchi (development in the absence of an inflammatory reaction of the bronchial wall). **(C)** ATHOS study synopsis. Exclusion criteria are the following: (i) cystic fibrosis-related bronchiectasis, (ii) traction’s bronchiectasis, (iii) previous lung and/or heart transplantation, and (iv) medical condition which could place patient at risk during exercise training (angina, neurological, or orthopedic condition). It is a pilot study, and a control group is not required. Study findings will be compared to storage data on bronchiectasis patients (with comparable features) who are categorized as frequent exacerbators in absence of subsequent active training programs.

Non-cystic fibrosis-related bronchiectasis (NCFB) is a chronic respiratory disease characterized by irreversible dilatation of the bronchial tree (Figures 1A,B). Clinically, its primary features are cough associated with excess mucus production, inflammation, and thickening of the airways resulting in poor ability to clear sputum. Epidemiologically, it is still a rare disease, despite the overall number of patients identified to be affected by this condition is increasing (9) and can be diagnosed by a high-resolution chest computed tomography scan. Acute exacerbations are a key driver in the progression of bronchiectasis and harm the natural history of the disease, leading to a worsened quality of life and higher socioeconomic burden. According to recent literature data, exacerbation frequency has been associated with worse clinical outcomes and increased mortality risk. Concerning this issue, it was observed that two or more exacerbations per year or one hospitalization/year were strong predictors of mortality among patients with bronchiectasis (2, 10, 11). been Much effort has been taken to validate the combination of clinical predictive variables that are able to identify patients at risk of mortality, hospitalization, and exacerbations across healthcare systems; among them, there is the Bronchiectasis Severity Index (BSI) (12). Antimicrobial administration consistency can strongly affect antibiotic resistance risks in the patient population, which is often chronically colonized. Past treatment errors caused a globally known health threat, known as multidrug-resistant (MDR) strains (13), which is seriously emerging as a health-care burden (14). Notably, NCFB is associated with diminished health-related quality of life (HRQoL) and reduced exercise tolerance (15). Although international guidelines for pulmonary rehabilitation recommend including patients with bronchiectasis (16, 17), there is a deficiency in evidence supporting exercise training in this population, and no athletic programs have been fully developed. Thus, it should be hypothesized that a specific athletic/physical program given to those NCFB patients with a higher performance status should be of help in improving their overall outcome.

## 2 Known evidence for exercise training in individuals with stable bronchiectasis

Bronchiectasis is characterized by excessive sputum production, chronic cough, and acute exacerbations and is associated with symptoms of dyspnea and fatigue, which reduce exercise tolerance and impair quality of life. Exercise training in isolation or in conjunction with other interventions is beneficial for people with other respiratory diseases, but its effects in bronchiectasis have not been well-established. Although existing literature suggests that exercise training, particularly when combined with co-interventions such as educational sessions and airway clearance therapy, can enhance functional exercise capacity and quality of life in individuals with stable bronchiectasis, the evidence supporting these benefits remains limited (18). Thus, low levels of physical activity and high sedentary time are associated with a higher risk of hospitalization for exacerbation (19). The rationale behind using exercise training in bronchiectasis management stems from the understanding of the disease’s impact on respiratory and peripheral skeletal muscles. Bronchiectasis often leads to reduced exercise tolerance and impaired physical function. Exercise training aims to address these issues by improving physical function, exercise tolerance, and respiratory symptoms. Moreover, studies on other chronic respiratory conditions, such as COPD (20, 21), have demonstrated the positive effects of endurance and strength training. These benefits include enhanced peripheral muscle strength and aerobic capacity, reduced dyspnea and fatigue, and improved quality of life (22). It is plausible that similar improvements could be observed in individuals with bronchiectasis, although the underlying mechanisms require further investigation. For people with stable bronchiectasis, evidence suggests that exercise training compared to usual care improves functional exercise tolerance as measured by the incremental shuttle walk distance; evidence also suggests that exercise training improves 6-min walk distance (6MWD, 1 study, 76 participants; low-certainty evidence). The magnitude of these observed mean changes appears clinically relevant as they exceed the minimal clinically important difference (MCID) thresholds for people with chronic lung disease. Evidence suggests that quality of life improves following exercise training according to St George’s Respiratory Questionnaire (SGRQ) total score (MD -9.62 points, 95% CI -15.67 to −3.56 points; 3 studies, 160 participants; low-certainty evidence), which exceeds the MCID of 4 points for this outcome. A reduction in dyspnea (MD 1.0 points, 95% CI 0.47 to 1.53; 1 study, 76 participants) and fatigue (MD 1.51 points, 95% CI 0.80 to 2.22 points; 1 study, 76 participants) was observed following exercise training according to these domains of the Chronic Respiratory Disease Questionnaire. However, there was no change in cough-related quality of life as measured by the Leicester Cough Questionnaire (LCQ) (MD -0.09 points, 95% CI -0.98 to 0.80 points; 2 studies, 103 participants; moderate-certainty evidence) nor in anxiety or depression. Two studies reported longer-term outcomes up to 12 months after intervention completion; however, exercise training did not appear to improve exercise capacity or quality of life more than usual care. Exercise training reduced the number of acute exacerbations of bronchiectasis over 12 months in people with stable bronchiectasis (odds ratio 0.26, 95% CI 0.08 to 0.81; 1 study, 55 participants). After an acute exacerbation of bronchiectasis, data from a single study (*N* = 27) suggest that exercise training compared to usual care confers little to no effect on exercise capacity (MD 11 m, 95% CI -27 to 49 m; low-certainty evidence), SGRQ total score (MD 6.34 points, 95%CI -17.08 to 29.76 points), or LCQ score (MD -0.08 points, 95% CI -0.94 to 0.78 points; low-certainty evidence) and does not reduce the time to first exacerbation (hazard ratio 0.83, 95% CI 0.31 to 2.22). These data are collected by a Cochrane review published in 2021 (16). This review provides low-certainty evidence suggesting improvement in functional exercise capacity and quality of life immediately following exercise training in people with stable bronchiectasis; however, the effects of exercise training due to inadequate reporting of methods, small study numbers, and variation between study findings, evidence is of very low-to-moderate certainty. Limited evidence is available to show longer-term effects of exercise training on these outcomes.

## 3 Aim and rationale of the study

As discussed above, rehabilitation is a fundamental part of treatment in bronchiectasis. Fewer data are instead available regarding a full training program based on athletic intervention reserved for highly performant patients, in the absence of comorbidity. We thus aim at validating an athletic training program, namely the athletic protocol in non-cystic fibrosis-related bronchiectasis (ATHOS) study, aiming at improving performances and quality of life. Patients will be selected based on their performance status. This approach assures the identification of patients presenting at lower risks for the program. The aim of the ATHOS study is to investigate the results obtained by administering a specific athletic/physical protocol to a cohort of patients who are diagnosed with bronchiectasis and are categorized as frequent exacerbators. Our hypothesis is to draw a clinical project to examine the short- and long-term effects of a 6-month outpatient exercise training program for patients affected by the NCFB bronchiectasis, where participants will be allocated to undergo a supervised exercise training program. We believe that it will improve both exercise capacity and quality of life and reduce the number of acute exacerbations in both the short and long term in people with bronchiectasis. To the best of our knowledge, this is the first perspective clinical trial encompassing an athletic program for NCFB patients. The main aim of the ATHOS trial is to set a high rate of aerobic capacity by progressive load with consequent improved systemic performance. The key player in the ATHOS trial is the patient, who will be in charge of evaluating the suitability of the proposed load. Moreover, the application of an athletic training program to the NFCB patient population aims at reducing the consumption of energy; in other words, amount of work measured in Joule, which is abnormally increased to adequate ventilatory performances in the context of this specific disease. In detail, it should be remarked that lung behavior should be considered to be elastic, meaning that a unique relation exists between the external force and the parenchymal deformation. This implies that the force versus stretch curves for the loading and unloading path (hysteresis) are identical. There is no history dependency, and all energy that is stored in the parenchymal during deformation is regained during the unloading phase. Consequently, the rate of loading or unloading does not affect the forces versus stretch curves. However, in the context of NCFB, the lung does not behave elastically. During the deformation cycle (ventilation), the force decreases in time, according to a phenomenon named relaxation, and a constant additional load is required, but ultimately it will increase the creep of the system. In NFCB, this viscoelastic behavior is pathologically altered, and this implies that energy is dissipated during ventilation. In this context, the increased required work load will be balanced by the empowered muscle mass. This will be of help in optimizing ventilation cycles and ultimately drainage of secretion.

## 4 Study design

**Figure 2:**
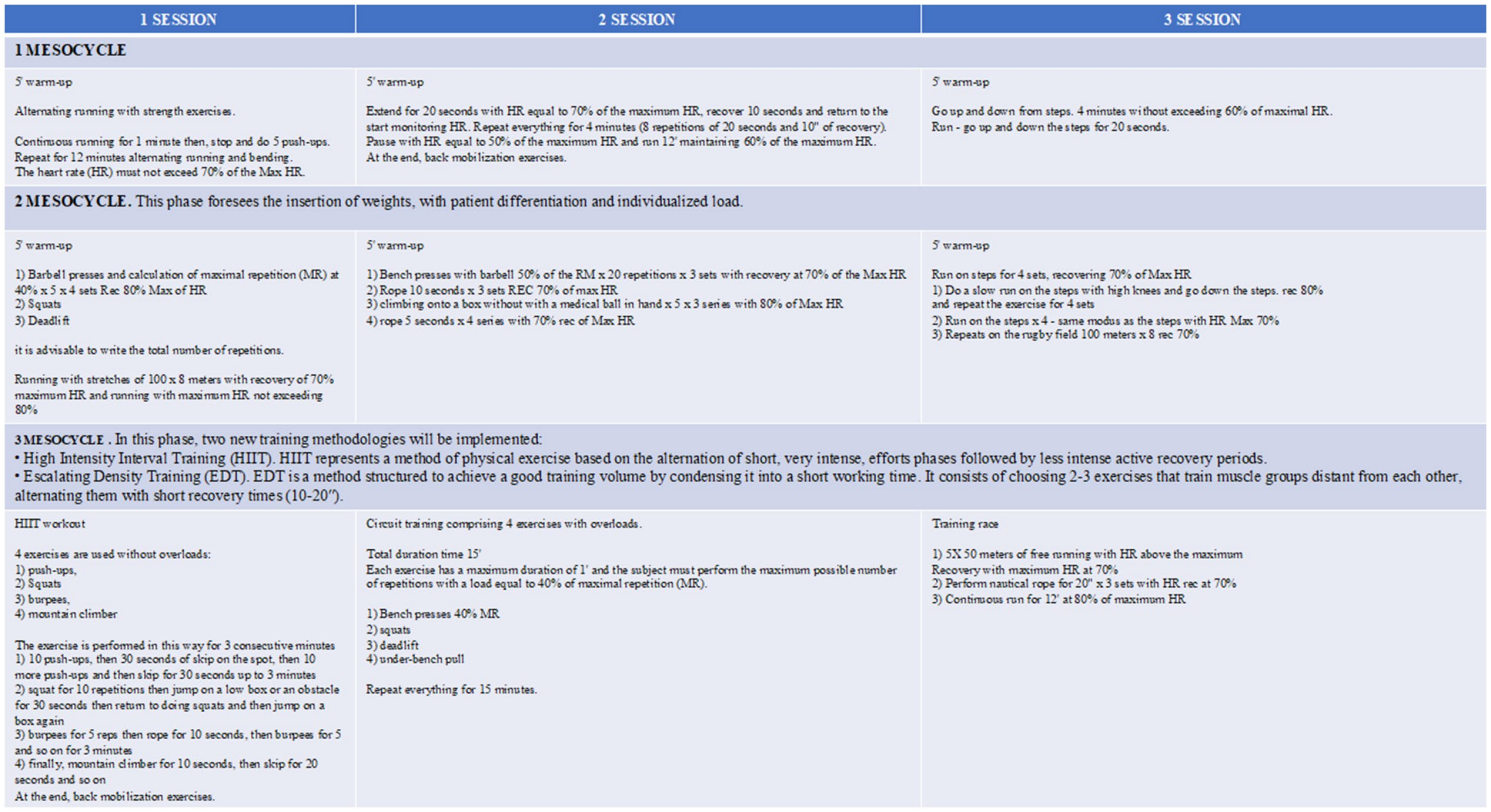
ATHOS training program. Each mesocycle will last 3 weeks. The theoretical maximum heart rate (HR), fundamental parameter for quantifying the intensity of training, will be calculated by Karvonen (32) formula. In case of subjects older than 65 years, the training program will be slightly modified.

Patients will be addressed for a work protocol based on mesocycles of 3 times/week for 6 months (Figure 2). A patient-tailored training regimen will be set up considering major complaints: endurance training for patients with dyspnea, strength training for patients with cough and difficult sputum expectoration, and a balanced aerobic and anaerobic training for patients with asthenia. Primary endpoints will be the reduction of acute disease exacerbations and hospital admissions and the improvement of patients’ QoL. Therefore, performing an interventional prospective study we aim at determining the effects of exercise training compared to usual care on: (i) incidence of acute exacerbation and hospitalization and the bronchiectasis severity index (BSI); (ii) exercise tolerance measured by cardiopulmonary exercise testing (CPET) maximum oxygen consumption (VO_2_ max) and distance covered at 6-min walking test; (iii) quality of life. Health-related quality of life will be assessed with St. George’s respiratory questionnaire (SGRQ); (iv) respiratory function measured by pulmonary function tests (PFTs) to measure lung volume, capacity, rates of flow, and gas exchange; (v) modified medical research council (MMRC) dyspnea scale; and (vi) improvement in caloric consumption measured by metabolic equivalents (METs). During the 8-week intervention period and over the 12-month follow-up, all participants will maintain a daily diary recording changes in symptoms. This symptom record will be used to identify an exacerbation, which was defined as the presence of ≥ four signs and symptoms (including change in sputum amount, thickness or color, hemoptysis, increased cough, tiredness, shortness of breath, or fever> 38°C) for two or more consecutive days with and without prescription of new antibiotics. To ensure adherence to diary completion, participants in both groups will be contacted by telephone monthly over the follow-up period. Exacerbation data were extrapolated from diary records by an independent assessor blinded to group allocation, which was then verified with the participant’s general practitioner or hospital records. The study synopsis is summarized in Figure 1C. To improve adherence to the intervention, each patient will be followed by a motor scientist during each phase of the active training program. Exercises will be shown, analyzed, and discussed before starting the program. Linear wire encoders, attached to the patient or the tool used, will calculate the amount of each movement and the speed at which they occur. Risks related to the program itself encompass muscular damage and bone fractures. To minimize the risk the enrolled subjects will be supervised by dedicated motor scientists during each step. This study will be conducted in compliance with the protocol, the current version of the Declaration of Helsinki, the ICH-GCP (good clinical practice), and all national legal and regulatory requirements and the local ethics commission.

## 5 Expected results from ATHOS trial

Exercise training holds promise as a valuable component of bronchiectasis management. Despite the theoretical basis for exercise training in bronchiectasis, the available evidence is limited by small sample sizes, mainly affected by cystic fibrosis (23–25) and a lack of long-term follow-up. Existing studies often suffer from methodological limitations, making it challenging to draw definitive conclusions about the efficacy of exercise training in this population. The ATHOS study is designed to address an unmet issue in NCFB patients since the proposed approach, in patients with good performance status and in early-stage disease, could represent a powerful tool to prevent and reduce infection and chronic colonization rates, ultimately leading to improved outcomes and quality of life of these patients. The ultimate goal is to allow the patient to understand that physical activity with ordered loads and personalized methodology would positively influence the quality of life in case of chronic disease, such as NCFB.
